# Genetic distance and heterogenecity between quasispecies is a critical predictor to IFN response in Egyptian patients with HCV genotype-4

**DOI:** 10.1186/1743-422X-4-16

**Published:** 2007-02-14

**Authors:** Abdel Rahman N Zekri, Hanaa M Alam El-Din, Abeer A Bahnassy, Mohsen M Khaled, Ashraf Omar, Inas Fouad, Mahmoud El-Hefnewi, Fouad Thakeb, Mostafa El-Awady

**Affiliations:** 1 Virology and Immunology Unit, Cancer Biology Department, National Cancer Institute, Cairo University.1^st ^Kasr El-Aini st, Cairo, Egypt; 2 Pathology Department, National Cancer Institute, Cairo University 1^st ^Kasr El-Aini st., Cairo, Egypt; 3 National Diabetes Institute, Ministry of Health, Egypt, Kasr El-Aini st., Cairo, Egypt; 4 Kasr El-Aini School of Medicine, Cairo University, Kasr El-Aini st., Cairo, Egypt; 5 National Research Center, Cairo, Egypt, Tahrir St., Cairo, Egypt

## Abstract

**Background:**

HCV is one of the major health problems in Egypt, where it is highly prevalent. Genotype 4 is the most common genotype of HCV and its response to treatment is still a controversy.

**Methods:**

HCV genotype 4 quasispecies diversity within the 5' untranslated region (5'UTR) was studied in a series of 22 native Egyptian patients with chronic hepatitis C virus with no previous treatment who satisfied all NIH criteria for combined treatment of pegylated IFN and ribavirine and was correlated with the outcome of treatment. The study also included 7 control patients with no antiviral treatment. HCV sequencing was done using the TRUGENE HCV 5-NC genotyping kit.

**Results:**

At the 48^th ^week of treatment, 15 patients (68%) showed virological response. Whereas HCV-RNA was still detected in 7 patients (32%) in this period; of those, 6 experienced a partial virological response followed by viral breakthrough during treatment. Only one patient did not show any virological or chemical response. The four females included in this study were all responders. There was a significant correlation between the response rate and lower fibrosis (p = 0.026) as well as the total number of mutation spots (including all the insertions, deletions, transitions and transversions) (p = 0.007, p = 0.035).

**Conclusion:**

Patients who responded to interferon treatment had statistically significant less number in both transitions (p = 0.007) and the genetic distances between the quasispecies (p = 0.035). So, viral genetic complexity and variability may play a role in the response to IFN treatment. The consensus alignment of all three groups revealed no characteristic pattern among the three groups. However, the G to A transitions at 160 was observed among non responders who need further study to confirm this observation.

## Background

Infection with hepatitis C virus (HCV) is a leading cause of chronic liver disease worldwide [1]. Despite recent success after the introduction of combination therapy with IFN-α and Ribavirin, about 60% of patients with HCV genotype 4 fail to respond [2,3]. Resistance to antiviral therapy remains a serious problem in the management of chronic hepatitis C.

The basis of treatment of chronic hepatitis C is interferon-α (IFN-α), which is currently used in combination with ribavirin, a molecule that potentiates its antiviral effects [4]. IFN-α does not inhibit a specific viral enzymatic function, but rather induces modifications of specific immune responses, and the establishment of a nonspecific antiviral state in infected cells by the activation of numerous cellular genes. Thus, the inhibition of HCV replication is a consequence, in part, of a global inhibition of translation in the infected cell. Therapeutic failure is frequent. The outcome of antiviral treatment seems to depend on many factors, among which virus-related parameters appear to play an important role [4]. These include the HCV genotype and the level of both the viral replication and the genetic complexity of the quasispecies population before the start of treatment [5,6].

Patients who do not have a sustained response to IFN therapy constitute a heterogeneous group [7]: some experience persistent viremia and alanine aminotransferase abnormalities (non-response) or a relapse after treatment discontinuation (transient response), whereas others have an initial response followed by reactivation while on INF therapy (breakthrough). About 10% of patients treated with IFN alone and 5% treated with IFN in combination with ribavirin experience a viral breakthrough during treatment [8,9]. Three main hypotheses have been proposed to explain viral breakthrough during IFN treatment: (1) the development of anti-IFN antibodies, (2) the down-regulation of IFN receptors (3) the emergence of resistant viral strains. The first hypothesis can be partially ruled out as anti-IFN antibodies are not consistently found in association with viral breakthrough [10,11]. Second, the down-regulation of IFN receptors during therapy, as observed for hepatitis B, has not been clearly correlated to treatment response [12]. The third hypothesis, which is the emergence of resistant strains during treatment, has been recently suggested [8]. And it was also shown that the evolution of hypervariable region 1 (HVR1) quasispecies during IFN mono-therapy was different between sustained responders and patients who experienced a viral breakthrough [13]. However, no longitudinal study has yet been performed on other regions within HCV genomes isolated from patients with a viral breakthrough. Further more little is known with respect to viral breakthrough during IFN-ribavirin combination therapy in Egyptian patients with HCV genotype 4.

The most prevalent genotype in Egypt is type 4, with the presence of other genotypes [14]. Sequence analysis of HCV 5'UTR in Egyptian patients with liver diseases showed a very highly heterogeneous population of the virus [15]. Therefore, we investigated heterogeneity, the composition and molecular evolution of HCV genotype 4 quasispecies during the course of IFN therapy by tracking individual viral variants in patients with chronic hepatitis C who exhibited different patterns of response. Our main objective was to determine whether viral genetic complexity and variability play a role in the response to IFN treatment in Egyptian HCV genotype-4 since no previous study have addressed or focused specially to Egyptian HCV genotype-4

## Patients and methods

The study included 22 subjects with anti-HCV positive and high levels of SGPT and SGOT for more than 6 months. They were selected from the outpatient clinic of El-Kaser El-Aini School of Medicine, Cairo University, Cairo, Egypt, during the period of October 2002 till November 2003. Of those 15 were responders, and 7 were non responders. The study also included 9 chronic hepatitis C patients who did not receive any antiviral treatment but received only conservative cytoprotective drugs. All cases were subjected to complete history taking and thorough clinical examination. Liver function tests and liver biopsy were performed. Samples were tested for anti-HCV using a commercially available EIA. (Satisfied NIH criteria for IFN and r TTT)

### Treatment strategy

Twenty two chronic hepatitis C patients received combined pegylated interferon alpha 2b (100 μg/week) plus ribavirin 800–1000 mg/day based on body weight (<70 kg or >70 kg). The other seven patients were considered as control group, and did not receive any antiviral treatment and received only conservative cytoprotective drugs.

### Inclusion criteria of our patients

Male or Female patients with chronic active hepatitis C virus, age from 18 to 60 years old negative for HBsAg and HBsAb, ANA<1:160. They were also positive for anti-HCV and HCV-RNA by PCR. All had white blood cells (WBCS)>4.000/mm^3^, Neutrophils count>2.000/mm^3^, Platelets>75.000/mm^3^, Prothrombin time<2 Seconds above upper limit of normal (ULN), Direct bilirubin 0.3 mg/dl or within 20% of ULN, Albumin>3.5, Alpha feto-protein<100, Serum creatinine within normal limit (WNL), Fasting blood sugar 115 mg or within 20% of ULN, also T3, T4 and TSH are within normal limit.

### Exclusion criteria of our patients

Exclusion of any other causes of liver disease other than HCV by liver biopsy including (Alpha 1-antitrypsin deficiency, Wilson's disease, Hemochromatosis). Exclusion of co-infection with HBV, Autoimmune disease, Alcoholic or decompensate liver disease, hypersensitivity to Interferon or Ribavirin, pregnancy or breast feeding, poorly controlled diabetes, clinically significant retinal abnormalities, obesity-induced liver disease, drug-related liver disease, CNS trauma, or active seizures which requires medication, ischemic cardiovascular disease within the last six months, Exclusion of immunological mediated disease (Ulcerative colitis, Crohn's disease, SLE, Autoimmune hemolytic anemia, Scleroderma, Psoriasis, Rheumatoid arthritis, patients with organ transplantation, past history of Bilhaziasis or substance abuse, abstention for the past 12 months, patients with Amantadine, Flumantadine, Thymosin, Steroids, and/or Immunosuppressive drugs, and sever pre-existing Psychiatric conditions or any history of manic element associated to prior Psychiatric history.

### Definition of response to therapy

The responses to therapy in patients with HCV were characterized according to the consensus guidelines of the National Institutes of Health [16]. Responders to therapy were defined by normalization of serum ALT and absence of detectable serum HCV-RNA at the end of treatment (12 months). Non-responders were defined by elevated serum ALT and presence of HCV-RNA at the end of treatment. Breakthrough was defined arbitrarily as an initial (after 6 months) biochemical and virological response, characterized by a normalization of ALT levels and a significant decrease of HCV-RNA titer of more than one log 10 up to no detectable HCV RNA, and a subsequent significant increase of more than one log 10 or reappearance of HCV-RNA in the serum during therapy, followed by return of ALT levels to abnormal values.

### Cloning and sequencing of the HCV 5-UTR region

For cloning and sequencing of the HCV 5-UTR, HCV RNA was extracted from the sera by the silica method as previously described [Boom et al., 1990]. RT and PCR of HCV were performed with a primer pair selected from the highly conserved 5-UTR of HCV genome [17]. All steps were done as previously described [18]. The following sequences were used as antisense primers for c-DNA synthesis HCV-6 [5-ACC-TCC nucleotides (NT) 319–324]. The internal primers were RB6A and RB6B for amplification of 266 bp of the 5-UTR, RB6A [5-GTG AGG AAC TAC TGT CTT CAC G-3 (NT 47–68)], and RB6B [5-ACT CGC AAG CAC CCT ATC AGG -3 (nt292-312)] [18]. All samples were analyzed twice for HCV RNA by the RT-PCR on different days with identical results. Upon completion of the amplification reaction, 10 μl of each PCR reaction product was analyzed by electrophoresis.

Molecular cloning was done with an Original TA cloning kit (Invitrogen Co., Carlsbad, CA). The PCR product was ligated into the pCR 2.1 vector and transformed into competent cells. Plasmid DNA was then amplified in *Escherichia coli *and purified with a Qiagen purification kit (Qiagen, Inc., Chatsworth, CA). Fifteen clones from each subject were analyzed. The clones were lettered Pre-T or Aft-T, and numbered from 1 to 15. The insert DNA was sequenced using the TRUGENE HCV 5-NC genotyping kit, Visible Genetics, Inc. (Toronto, Ontario, Canada) was used in conjunction with the Open Gene DNA sequencing system. A positive control of known standard sequence and a negative HCV control provided with the kit were utilized as controls in each run and sequenced by CLIP sequencing which allows both directions of the target amplicon to be sequenced simultaneously in the same tube using two different dye-labeled primers (Cy5.0 and Cy5.5) for each reaction. This method provides sequence information for both positive and negative DNA strands from a single reaction. The forward and reverse sequences are combined to form a query sequence. The query sequence is then compared to previously characterized isolates in the TRUGENE HCV 5- NC Module of the Open Gene software system in order to determine the HCV genotype of the sample. Gene Objects software analyzes chromatograms from each sample; the final 5- UTR sequence was obtained from the comparison of both sequenced strands [15]. This information is compared with deposited HCV sequences by Gene Librarian TM software with a minimal concordance of 98%. The genotype assignments of these samples were confirmed by BLAST searches.

### Multiple sequence alignment

The sequences of the three groups were aligned using the Clustalw 1.81 software [19]. Sequence alignment editing visualization, and conservation, and positional entropy plots were done on BIOEDIT V 7.0 [20]. The positional entropy plot is a measure of the lack of predictability for an alignment position. The entropy is 0 at a position where you have a maximum guess (information) for nucleotide sequence in this position, and it is maximum when you have equal possibility for each nucleotide (A, G, C or T) in this position. Analysis of insertions, deletions, transitions (in which a purine is substituted for a purine or a pyrimidine for a pyrimidine), and transversions (in which a purine is substituted for a pyrimidine and vice versa) was done on the sequence alignment of all groups against the consensus of each group. Phylogenetic Analysis and distances of the sequences was done using Clustalw 1.81 software.

### Statistical analysis

The results are expressed as the mean ± SD. Non-parametric ANOVA (Kruskal-Wallis test) was used to compare means of more than 2 groups. Significance levels of ≤ 0.05 were considered significant.

### Nucleotide sequence accession numbers

The nucleotide sequences reported here are deposited in The National Center for Biotechnology Information/National Institute of Health GenBank nucleotide sequence database The accession numbers are: [GenBank: AY 661552, AY 673080–AY 673111].

## Results

Distribution of different HCV genotypes in the studied groups is shown in table 1. As proven by Blast analysis, genotype 4 was the most common genotype, representing 90% of the cases, the rest three cases (10%) had genotypes 1a, 1b, and 3a. All of the responders were infected with genotype 4, except two patients with genotype 1b and 3a. Also one of the nonresponders was infected with HCV genotype 1a (breakthrough patient number 6).

**Table 1:** HCV genotypes in the different study groups

Genotype	Responders (n = 15)	Non-responders (n = 7)	Untreated controls (n = 9)
1a	-	1	-
1b	1	-	-
3a	1	-	-
4	8	3	7
4a	5	2	1
4g	-	1	1

At the 48^th ^week of treatment, HCV RNA was cleared from serum in 15 responder patients (68%) whereas HCV RNA was still positive in the serum of 7 non responder patients (32%) in this period; 6 of those 7 patients experienced a viral breakthrough during treatment. HCV RNA dropped to undetectable levels in 3 and in 3 patients HCV RNA decreased but did not become undetectable.

The three groups of patients were similar with respect to demographic and clinical characteristics except that the total population was male and the four females included in this study were all responders. Also, responders were characterized by a less fibrosis score in the liver biopsy than non responders, and the difference was statistically significant (p = 0.026) (Table 2).

**Table 2:** Factors playing a role in the effectiveness of interferon therapy in patients with chronic hepatitis C according to their response to IFN-α therapy and in untreated controls.

Factor	Responders (n = 15)	#Non-responders (n = 7)	untreated controls (n = 9)	*P *value*
Age (mean ± SD)	42 ± 6.8	42.5 ± 3.2	43.6 ± 4.5	0.78
M:F	11:4	7:0	9:0	0.15
Mean Weight	83.3 ± 13.6	90 ± 11.9	87.2 ± 8.7	0.562
**Blood picture:**				
WBCs	7 ± 2.4	6.3 ± 1.9	5.3 ± 1	0.119
HGB	14.4 ± 1.7	15 ± 1.2	14.4 ± 1	0.495
PLT	208.9± 55.7	187.6 ± 49.2	175.2 ± 43.1	0.448
**Liver function tests:**				
AST	91.1 ± 46.6	63.1 ± 26.6	70.2 ± 29.5	0.282
ALT	140 ± 111	83.5 ± 45.2	92.5 ± 43.4	0.361
Albumin	4.57 ± 0.39	4.46 ± 0.46	4.48 ± 0.43	0.836
PT	12.0 ± 0.65	12.2 ± 0.87	12.1 ± 0.77	0.953
INR	1.07 ± 0.1	1.1 ± 0.13	1.07 ± 0.1	0.856
Iron	117 ± 37.5	129 ± 34	117 ± 35	0.812
Ferritin	311 ± 243	504 ± 864	444 ± 760	0.702
**Kidney function tests:**				
Urea	28.5 ± 6.7	28.7 ± 4.6	29.6 ± 5.9	0.959
Creatinine	0.88 ± 0.18	0.91 ± 0.17	0.87 ± 0.15	0.807
**Liver echogenicity:**				
HAI	5.6 ± 2.7	6.7 ± 3.4	5.8 ± 2.6	0.764
Fibrosis score	1.1 ± 0.9	2.9 ± 1.8	2.1 ± 1.6	0.026*

# Non-responders include I non responder and 6 viral breakthrough patients

* P value is significant between responder and non-responders

**Figure 1:**
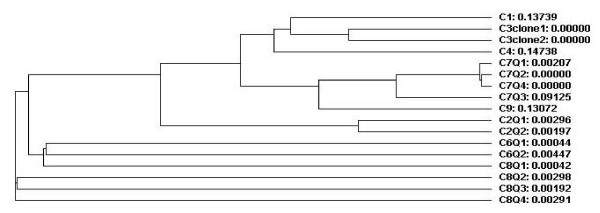
Phylogenetic trees of 5' noncoding region quasispecies in control group; before IFN treatment and at the end of follow up, i.e., 48 weeks after IFN withdrawal.

**Figure 2:**
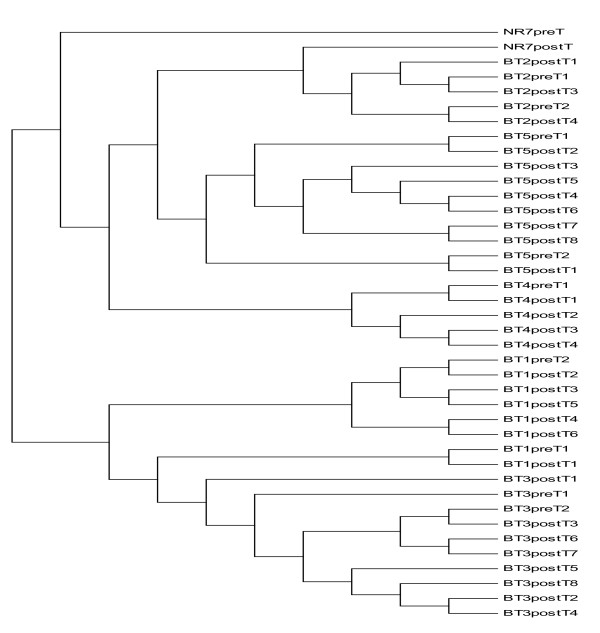
Phylogenetic trees of 5' noncoding region quasispecies in non responder group; before IFN treatment and at the end of follow up, i.e., 48 weeks after IFN withdrawal.

**Figure 3:**
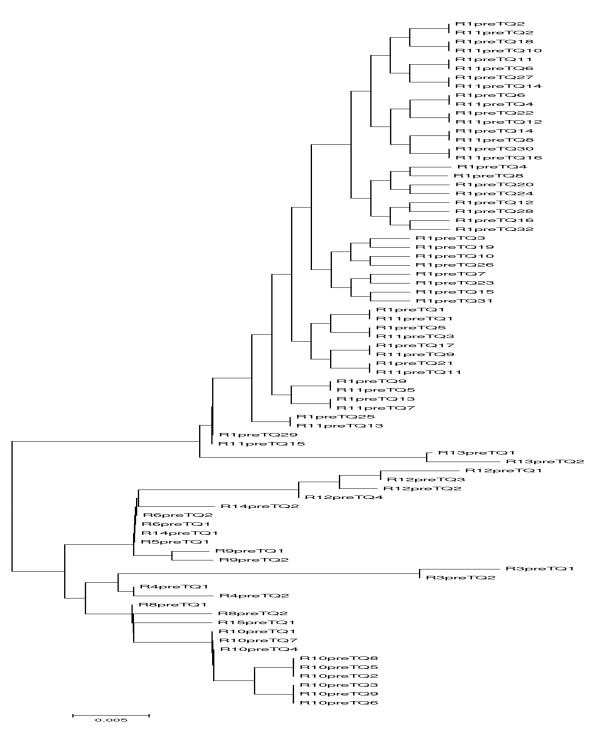
Phylogenetic trees of 5' noncoding region quasispecies in responder group; before IFN treatment and at the end of follow up, i.e., 48 weeks after IFN withdrawal.

**Figure 4:**
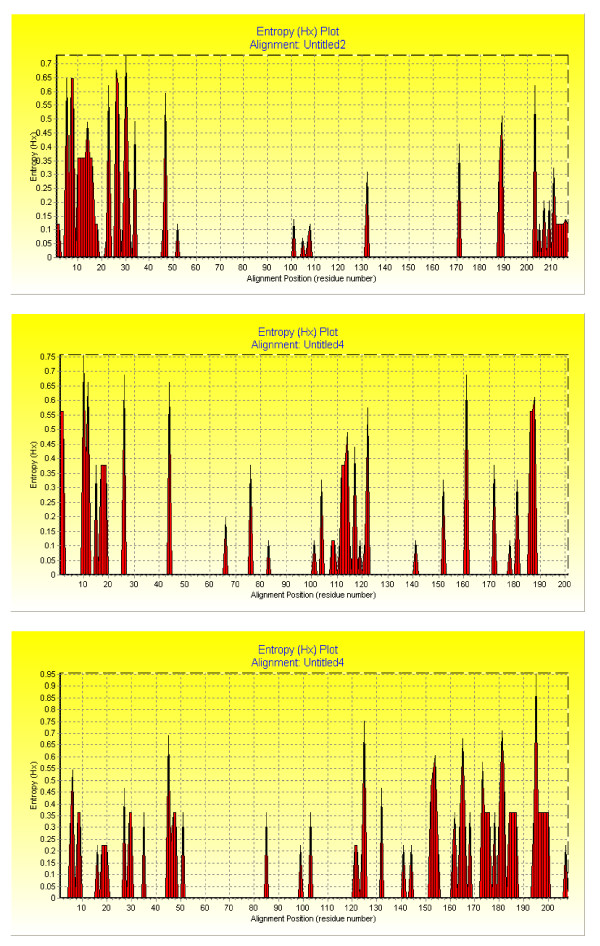
Position entropy plots for quasispecies sequences of (a) responder group (b) nonresponder group (c) control group.

The mean base line viral load and number of quasispecies was higher in the responder group compared to the nonresponders and controls, however, the difference was not statistically significant (Table 3). Whereas patients who responded to interferon treatment had statistically significant (p = 0.007, p = 0.035) less number of transitions and genetic distances between the quasispecies than in the other two groups (Table 3). (see Additional file 1 a–d). The genetic distances were made available through the phylogenetic trees of controls group (Figure 1), nonresponders and breakthrough patients (figure 2) and responders (figure 3). Three breakthrough patients cleared the virus after three months but returned to nearly the same base line viral load. The other three breakthrough patients had a mean of 2.5 folds less viral load by the third month and returned to the same baseline viral load. Table 3 also shows that the total number of mutation spots (including all the insertions, deletions, transitions and transversions) were less among responders than in the other two groups (p = 0.275). This was also obvious by their position entropy plots (figure 4) which showed that responders had the lowest number of position variability than among the other two groups.

**Table 3:** Viral load, and quasispesies variability in different study groups

Factor	Responders (n = 13)	#Non-responders (n = 6)	untreated controls (n = 8)	*P *value*
Base line viral load: **(mean ± SD)	661.8 ± 1286.7	391.3 ± 201.4	329.4 ± 183.1	0.719
No of quasispecies: Median (range)	2.0 (1–32)	2.0 (1–8)	1.0 (1–4)	0.262
Sequence diversity***:				
Insertions:	1.08 ± 1.9	0.33 ± 0.52	1.5 ± 1.7	0.345
Deletions :	1.2 ± 0.59	0.66 ± 0.82	1.1 ± 0.83	0.304
Transitions:	1.8 ± 0.9	4.7 ± 2.0	7.0 ± 6.8	0.007*
Tnasversions:	2.0 ± 2.5	2.5 ± 2.1	4.3 ± 4.5	0.482
Transition/Transversion:	1.3 ± 1.1	2.4 ± 1.2	2.3 ± 3.2	0.183
No. of mutation spots	6.2 ± 3.1	8.2 ± 4.2	13.9 ± 11.1	0.275
Genetic distance	0.005 ± 0.24	0.14 ± 0.73	0.19 ± 0.48	0.035*

# Non-responders include I non responder and 6 viral breakthrough patients, * P value is significant between responder and non-responders, **Viral load in thousands *** (mean ± SD)

We constructed a consensus sequence of each group that shows the highest expectancy of bases in every position. The consensus alignment of all three groups revealed no characteristic pattern during viral breakthrough and controls, whose genome sequences looked very unsimilar in their alignments (see Additional file 1a–d).

Table 4 shows the conserved and variable regions in the sequences of the three studied groups. It was noticed that responders are characterized by larger conserved sequence regions in comparison to the nonresponders and controls. Sequences from 35 to 100 and from 100–170 were highly conserved among responders except in patient number 10 who showed transition at position 47, and patients numbers 3, 4, and 9 who had variable position 70. The highest variability is between positions 5 and 34.

**Table 4:** Conserved and variable regions of HCV sequences among the three gropus

Group	Conserved regions	Variable regions
Responders	35–45, 53–99, 109–131, 133–170, 172–187	5–34
Nonresponders	3–9, 27–43, 45–65, 67–75, 84–100, 123–140	10–19, 108–122
Controls	1–4, 10–15, 21–26, 36–44, 52–84, 86–98, 104–120	161–187

Sequence region from nucleotide 35 to 100 of the pretherapeutic sequences were highly conserved between responders and the nonresponder and breakthrough patients infected by the same genotype, and remained highly conserved during and after treatment in all patients except for the nonresponder patient (3 positions), breakthrough number 3 (2 positions), and number 4 (1position).

The patient who did not show any response throughout the whole treatment period had a one insertion at position between 187–189, whereas it showed 8 transitions, and 5 transversions after treatment that were not there in the before treatment. Also breakthrough patient number 1 had only one transition before treatment but had three transitions and 1 transversion at the end of treatment. Generally, the total number of mutational spots in the nonresponders before treatment is less than that at the end of treatment (Table 5).

**Table 5:** Quasispecies diversity in nonresponders before and after treatment

	insertions	Deletions	Transition	Transversion	Total (number of mutational spots)	Genotype	Number of quasispecies
NR pre	1	0	0	0	1	4g	1
NR post	0	0	8	5	13	4g	1
BT1 pre	0	0	1	0	1	4a	2
BT1 post	0	0	3	1	4	4a	6
BT2 pre	0	2	6	3	11	4	2
BT2post	0	2	6	4	12	4	4
BT3 pre	0	1	2	1	4	4a	2
BT3 post	0	1	4	1	8	4a	8
BT4 pre	0	1	1	0	2	4	1
BT4 post	0	1	3	0	4	4	4
BT5 pre	1	0	4	1	6	4	2
BT5 post	1	0	4	4	9	4	8

**Figure 5:**
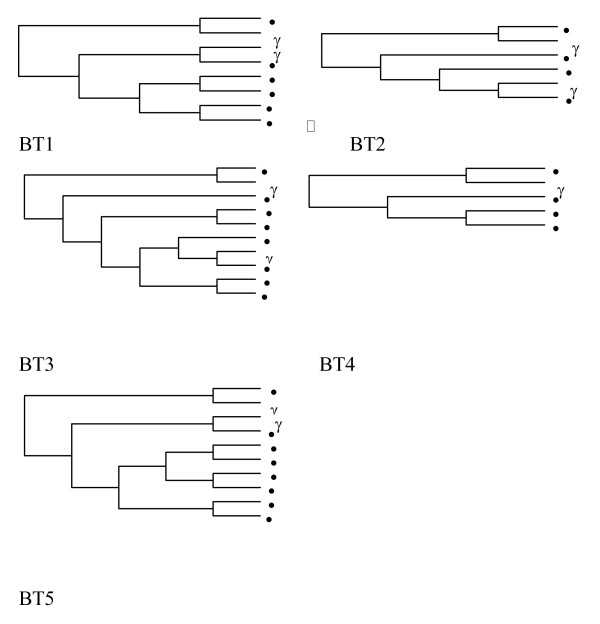
Phylogenetic trees of 5' noncoding region quasispecies in the 5 subjects studied before IFN treatment and at the end of follow up, i.e., 48 weeks after IFN withdrawal. The phylogenetic reconstructions are neigbour-joining trees, pretreatment sequences are presented by filled circles and post-treatment sequences are presented by open circles.

Table 5 and the alignment in additional file 1-b also shows that after treatment with interferon, the sequence showed mutations compared to the pretherapeutic samples in NR, BT1, BT3, BT4, and BT5. Figure 5 shows the phylogenetic tree of those patients individually pre and post treatment with no great differences among each. The only specific mutation identified is a transition from "G" to "A" in BT2 and BT5 pre treatment that was also mutated in the NR (genotype 4g), BT2, BT4, and BT5 (all genotype 4) after treatment. This position is not mutated in the responder group.

## Discussion

Antiviral treatment of HCV with interferon (IFN) α, or more recently pegylated IFN α, and ribavirin lead to a sustained virological response in more than 50 % of patients [21-23]. Patients with chronic hepatitis due to hepatitis C virus type 4 showed 8% sustained virological response to interferon alone but 52% response rates to interferon combined with ribavirin [24]. When Pegylated interferon/ribavirin was used to treat chronic hepatitis C genotype 4 patients, 13/30 (43.3%) showed end of treatment virological response in comparison to 11/31 (35.5%) only after conventional interferon/ribavirin combination therapy [25]. Their figure was less than ours; as our patients with genotype 4 showed sustained response rates of 68% when treated with pegylated interferon/ribavirin combination treatment for 48 weeks. The differences noticed might be due to the fact that their patients had compensated liver diseases; unlike ours. This figure, however, was in agreement with that in our previous study [26].

The current study showed that all female patients responded to treatment, which confirms the results of Alves et al. [27] who stated that combination therapy with interferon-alpha plus ribavirin was effective in one third of patients. Higher rates of response were observed in women and in patients infected by genotypes other than 1. This was in contrast with the study of Fargion et al. [28] in which no significant association with response was found with sex or age. Also our previous study showed that six out of 9 (67%) females treated with standard interferon had sustained viral response, whereas only one of 5 females treated with pegylated interferon showed viral response [26].

In this study, the fibrosis score negatively affected the response of HCV patients to PegIFN treatment. This was in concordance with Garrido et al. [29] who stated that Knodell's index like absence of fibrosis at liver histopathology had a predictive value of response, as opposed to gender, γGT level and source of infection. But that was against Fargion et al. [28] who found no association of response to IFN treatment with Knodell score.

The current study elucidated that pretreatment viral load did not affect the outcome of treatment, which agrees with the results of Fallows et al. [30], in which HCV RNA levels did not differ between IFN alpha responders and nonresponders. However, Magrin et al. [31] found in their study on 100 patients with HCV-RNA positive chronic liver disease that, in absence of cirrhosis, low pre-treatment serum HCV-RNA level is the most important predictor of response to IFN therapy.

Non-response is currently defined by the detection of HCV RNA in serum at the end of treatment. Schematically, two types of profiles can be observed: the absence of response during treatment or an initial decrease of HCV RNA levels followed by an increase or reappearance of viraemia, also called viral breakthrough [8].

Our study provided evidence that the number of viral strains and the genetic diversity before treatment did not correlate with treatment outcome which is in agreement with Farci et al. [13]. Consistent with these observations, phylogenetic analysis of viral sequences obtained from all patients before treatment failed to show any clustering associated with a specific pattern of response. In contrast, within the limitation of our data set, analysis of the early evolution of the viral quasispecies yielded important prognostic information. In patients who exhibited a sustained therapeutic response, we documented a significant decrease in the number of viral strains, and overall genetic diversity. A dramatic reduction in genetic diversity leading to an increasingly homogeneous viral population was a consistent feature associated with viral clearance in sustained responders and was independent of HCV genotype [32]. Also in agreement with our results, a strikingly similar trend of decreasing viral diversity was recently documented just before viral clearance in acute resolving hepatitis, whereas an increase in viral diversity was found to correlate with acute hepatitis that progressed to chronicity [32].

Regardless of the mechanism, it is surprising that an RNA virus that apparently does not induce latent infection can persist for weeks to months, in both breakthrough and relapsed patients, in the face of a seemingly complete suppression of viremia. The most plausible hypothesis is that, despite the disappearance of viremia, very low levels of viral replication continue to occur, most likely in the liver, providing a persistent reservoir for virus reactivation after relapse from the suppressive effects of IFN [32].

The analysis of HCV quasispecies revealed no characteristic pattern during treatment in breakthrough patients, whose HCV genome profile looked most similar to that of non-responders [8]. This was evident in our results by comparing the consensus of the three groups with no apparent difference in their patterns. Our findings showed that nucleotide 35–100 is highly conserved between the three studied groups. The 5'UTR is highly conserved out of the HCV genomic organization [33].

Analysis of Egyptian genotype 4 in this study was found to be characterized by a unique mutation site at The G (160) → A transition which is a unique mutation among non responders.

In conclusion, it seems that in HCV genotype 4 in Egypt, Phylogenetic analysis of viral sequences obtained failed to show any clustering associated with a specific pattern of response. The consensus alignment of all three groups revealed no characteristic pattern among the three groups. The G (160) → A is a unique mutation among non responders. There is an apparent reduction in genetic diversity leading to an increasingly homogeneous viral population associated with viral clearance in responders.

## Abbreviations

5' untranslated region (5'UTR), interferon (IFN), the **hypervariable region 1 (HVR1)**, National Institutes of Health (NIH), Ribavirin treatment (r TTT), Kilogram (Kg), Antinuclear antibody (ANA), Total leucocytivc count (WBCs), Within normal limit (WNL), Upper limit of normal (ULN), Central nervous system (CNS), Systemic lupus erythromatosis (SLE), Alanine transaminase (ALT), standard deviation (SD), Non responder (NR), Breakthrough (BT), Gamma Glutamide transferase (γ GT)

## Competing interests

The author(s) declare that they have no competing interests.

## Authors' contributions

ARNZ: Conducted all the practical part of the experiment, entitled the paper, and coordinate the whole work team. HMAED: Helped in the practical part and wrote the menauscript and edited the paper. AAB: Helped in the practical part, diagnosed all the histopathology samples for the patients, and wrote the namuscript. MOK and AO: Clinician responsible for the treatment policy. IF: sample collection MEH: shared in the biostatistics FT: Coordinated the research effort. MEA: Shared in biostatistics analysis and sequence analysis. All eight co-authors read and approved the final manuscript.

## Supplementary Material

Additional File 1 Sequence alignments of 5' non coding region quasispecies in (a) responder patient group, (b) non responder group, (c) control group (d) Consensus of the three studied groups. The data provided represent the sequence alignments of the three studied groups before IFN treatment and at the end of follow up, i.e., 48 weeks after IFN withdrawal. Click here for file
