# Supplementary material for: Genetic distance and heterogenecity between quasispecies is a critical predictor to IFN response in Egyptian patients with HCV genotype-4
Source: Virol J. 2007 Feb 14;4:16. doi: 10.1186/1743-422X-4-16 (PMC1805740; doi:10.1186/1743-422X-4-16)
Supplement: Additional File 1 — Sequence alignments of 5' non coding region quasispecies in (a) responder patient group, (b) non responder group, (c) control group (d) Consensus of the three studied groups. The data provided represent the sequence alignments of the three studied groups before IFN treatment and at the end of follow up, i.e., 48 weeks after IFN withdrawal. [file 1743-422X-4-16-S1.doc]

....|....| ....|....| ....|....| ....|....| ....|....| ....|....|

5 15 25 35 45 55

Consensus GCCATGGCG- ------TTAG TATGATGTGT TGTACAGCCT CCAGGACCCC CCTCCCGGGA

R1preT Q1 ....G.CA.- ------.... ..A...A..A .......... .......... ..........

R1preT Q2 ....G.CA.- ------.... ..A...A..A .......... .......... ..........

R1preT Q3 ....G.CA.- ------.... ..A...A..A .......... .......... ..........

R1preT Q4 ....G.CA.- ------.... ..A...A..A .......... .......... ..........

R1preT Q5 ....G.CA.- ------.... ..A...A... .......... .......... ..........

R1preT Q6 ....G.CA.- ------.... ..A...A... .......... .......... ..........

R1preT Q7 ....G.CA.- ------.... ..A...A... .......... .......... ..........

R1preT Q8 ....G.CA.- ------.... ..A...A... .......... .......... ..........

R1preT Q9 ....G.CA.- ------.... ..A......A .......... .......... ..........

R1preT Q10 ....G.CA.- ------.... ..A......A .......... .......... ..........

R1preT Q11 ....G.CA.- ------.... ..A......A .......... .......... ..........

R1preT Q12 ....G.CA.- ------.... ..A......A .......... .......... ..........

R1preT Q13 ....G.CA.- ------.... ..A....... .......... .......... ..........

R1preT Q14 ....G.CA.- ------.... ..A....... .......... .......... ..........

R1preT Q15 ....G.CA.- ------.... ..A....... .......... .......... ..........

R1preT Q16 ....G.CA.- ------.... ..A....... .......... .......... ..........

R1preT Q17 ....G.CA.- ------.... ......A..A .......... .......... ..........

R1preT Q18 ....G.CA.- ------.... ......A..A .......... .......... ..........

R1preT Q19 ....G.CA.- ------.... ......A..A .......... .......... ..........

R1preT Q20 ....G.CA.- ------.... ......A..A .......... .......... ..........

R1preT Q21 ....G.CA.- ------.... ......A... .......... .......... ..........

R1preT Q22 ....G.CA.- ------.... ......A... .......... .......... ..........

R1preT Q23 ....G.CA.- ------.... ......A... .......... .......... ..........

R1preT Q24 ....G.CA.- ------.... ......A... .......... .......... ..........

R1preT Q25 ....G.CA.- ------.... .........A .......... .......... ..........

R1preT Q26 ....G.CA.- ------.... .........A .......... .......... ..........

R1preT Q27 ....G.CA.- ------.... .........A .......... .......... ..........

R1preT Q28 ....G.CA.- ------.... .........A .......... .......... ..........

R1preT Q29 ....G.CA.- ------.... .......... .......... .......... ..........

R1preT Q30 ....G.CA.- ------.... .......... .......... .......... ..........

R1preT Q31 ....G.CA.- ------.... .......... .......... .......... ..........

R1preT Q32 ....G.CA.- ------.... .......... .......... .......... ..........

R3preT Q1 .........- ------.... .....-..TG ...G...... .....--... ..........

R3preT Q2 .........- ------.... .....-..TG ...G...... .....--... ..........

R4preT Q1 .........- ------.... .....-.... .......... .......... ..........

R4preT Q2 .........- ------.... .....-.... .......... .......... ..........

R5preT Q1 .........- ------.... .....-.... .......... .......... ..........

R6preT Q1 .........- ------.... .....-.... .......... .......... .-........

R6preT Q2 .........- ------.... .....-.... .......... .......... .-........

R8preT Q1 .........- ------.... .....-.... ...G...... ......-... ..........

R8preT Q2 .........- ------.... .....-.... .A.G...... ......-... ..........

R9preT Q1 .........- ------.... .....-.... .......... .......... ..........

R9preT Q2 .........- ------.... .....-.... .......... .......... ..........

R10preT Q1 .........A TAAAGG.... .....-.... A..G...... ......T... ..........

R10preT Q2 .........A TAAAGG.... .....-.... A..G...... ......T... ..........

R10preT Q3 .........A TAAAGG.... .....-.... A..G...... ......T... ..........

R10preT Q4 .........A TAACGG.... .....-.... A..G...... ......T... ..........

R10preT Q5 .........A TAACGG.... .....-.... A..G...... ......T... ..........

R10preT Q6 .........A TAACGG.... .....-.... A..G...... ......T... ..........

R10preT Q7 .........A TAAGGG.... .....-.... A..G...... ......T... ..........

R10preT Q8 .........A TAAGGG.... .....-.... A..G...... ......T... ..........

R10preT Q9 .........A TAAGGG.... .....-.... A..G...... ......T... ..........

R11preT Q1 ....G.CA.- ------.... ..A..-A..A .......... .......... ..........

R11preT Q2 ....G.CA.- ------.... ..A..-A..A .......... .......... ..........

R11preT Q3 ....G.CA.- ------.... ..A..-A... .......... .......... ..........

R11preT Q4 ....G.CA.- ------.... ..A..-A... .......... .......... ..........

R11preT Q5 ....G.CA.- ------.... ..A..-...A .......... .......... ..........

R11preT Q6 ....G.CA.- ------.... ..A..-...A .......... .......... ..........

R11preT Q7 ....G.CA.- ------.... ..A..-.... .......... .......... ..........

R11preT Q8 ....G.CA.- ------.... ..A..-.... .......... .......... ..........

R11preT Q9 ....G.CA.- ------.... .....-A..A .......... .......... ..........

R11preT Q1 ....G.CA.- ------.... .....-A..A .......... .......... ..........

R11preT Q1 ....G.CA.- ------.... .....-A... .......... .......... ..........

R11preT Q1 ....G.CA.- ------.... .....-A... .......... .......... ..........

R11preT Q1 ....G.CA.- ------.... .....-...A .......... .......... ..........

R11preT Q1 ....G.CA.- ------.... .....-...A .......... .......... ..........

R11preT Q1 ....G.CA.- ------.... .....-.... .......... .......... ..........

R11preT Q1 ....G.CA.- ------.... .....-.... .......... .......... ..........

R12preT Q1 .....A...- ------.... .....-.... .......... .......... ..........

R12preT Q2 .....A...- ------.... .....-.... .......... .......... ..........

R12preT Q3 .........- ------.... .....-.... .......... .......... ..........

R12preT Q4 .........- ------.... .....-.... .......... .......... ..........

R13preT Q1 ....GCCA.- ------GC.. .....-.... .......... .......... ..........

R13preT Q2 ....GCCA.- ------GC.. .....-.... ...G...... .......... ..........

R14preT Q1 .........- ------.... .....-.... .......... .......... ..........

R14preT Q2 .........- ------.... .....-.... .......... .......... ..........

R15preT Q1 .........- ------.... .G...-.... ...G...... ......-... ..........

....|....| ....|....| ....|....| ....|....| ....|....| ....|....|

65 75 85 95 105 115

Consensus GAGCCATAGT GGTCTGCGGA ACCGGTGAGT ACACCGGAAT CGCCGG--GA TGACCGGGTC

R1preT Q1 .......... .......... .......... .......... ......--.. ..........

R1preT Q2 .......... .......... .......... .......... ......--.. ..........

R1preT Q3 .......... .......... .......... .......... ......--.. ..........

R1preT Q4 .......... .......... .......... .......... ......--.. ..........

R1preT Q5 .......... .......... .......... .......... ......--.. ..........

R1preT Q6 .......... .......... .......... .......... ......--.. ..........

R1preT Q7 .......... .......... .......... .......... ......--.. ..........

R1preT Q8 .......... .......... .......... .......... ......--.. ..........

R1preT Q9 .......... .......... .......... .......... ......--.. ..........

R1preT Q10 .......... .......... .......... .......... ......--.. ..........

R1preT Q11 .......... .......... .......... .......... ......--.. ..........

R1preT Q12 .......... .......... .......... .......... ......--.. ..........

R1preT Q13 .......... .......... .......... .......... ......--.. ..........

R1preT Q14 .......... .......... .......... .......... ......--.. ..........

R1preT Q15 .......... .......... .......... .......... ......--.. ..........

R1preT Q16 .......... .......... .......... .......... ......--.. ..........

R1preT Q17 .......... .......... .......... .......... ......--.. ..........

R1preT Q18 .......... .......... .......... .......... ......--.. ..........

R1preT Q19 .......... .......... .......... .......... ......--.. ..........

R1preT Q20 .......... .......... .......... .......... ......--.. ..........

R1preT Q21 .......... .......... .......... .......... ......--.. ..........

R1preT Q22 .......... .......... .......... .......... ......--.. ..........

R1preT Q23 .......... .......... .......... .......... ......--.. ..........

R1preT Q24 .......... .......... .......... .......... ......--.. ..........

R1preT Q25 .......... .......... .......... .......... ......--.. ..........

R1preT Q26 .......... .......... .......... .......... ......--.. ..........

R1preT Q27 .......... .......... .......... .......... ......--.. ..........

R1preT Q28 .......... .......... .......... .......... ......--.. ..........

R1preT Q29 .......... .......... .......... .......... ......--.. ..........

R1preT Q30 .......... .......... .......... .......... ......--.. ..........

R1preT Q31 .......... .......... .......... .......... ......--.. ..........

R1preT Q32 .......... .......... .......... .......... ......--.. ..........

R3preT Q1 .......... .......... .......... .......... T.....--.. ..........

R3preT Q2 .......... .......... .......... .......... ......--.. ..........

R4preT Q1 .......... .......... .......... .......... ......-G.. ..........

R4preT Q2 .......... .......... .......... .......... ....A.TG.. ..........

R5preT Q1 .......... .......... .......... .......... ......--.. ..........

R6preT Q1 .......... .......... .......... .......... ......--.. ..........

R6preT Q2 .......... .......... .......... .......... ......--.. ..........

R8preT Q1 .......... .......... .......... .......... ......--.. ..........

R8preT Q2 .......... .......... .......... .......... ......--.. ..........

R9preT Q1 .......... .......... .......... .......... ......--.. ..........

R9preT Q2 .......... .......... .......... .......... ......--.. ..........

R10preT Q1 .......... .......... .......... .......... ......--.. ..........

R10preT Q2 .......... .......... .......... .......... ......--.. ..........

R10preT Q3 .......... .......... .......... .......... ......--.. ..........

R10preT Q4 .......... .......... .......... .......... ......--.. ..........

R10preT Q5 .......... .......... .......... .......... ......--.. ..........

R10preT Q6 .......... .......... .......... .......... ......--.. ..........

R10preT Q7 .......... .......... .......... .......... ......--.. ..........

R10preT Q8 .......... .......... .......... .......... ......--.. ..........

R10preT Q9 .......... .......... .......... .......... ......--.. ..........

R11preT Q1 .......... .......... .......... .......... ......--.. ..........

R11preT Q2 .......... .......... .......... .......... ......--.. ..........

R11preT Q3 .......... .......... .......... .......... ......--.. ..........

R11preT Q4 .......... .......... .......... .......... ......--.. ..........

R11preT Q5 .......... .......... .......... .......... ......--.. ..........

R11preT Q6 .......... .......... .......... .......... ......--.. ..........

R11preT Q7 .......... .......... .......... .......... ......--.. ..........

R11preT Q8 .......... .......... .......... .......... ......--.. ..........

R11preT Q9 .......... .......... .......... .......... ......--.. ..........

R11preT Q1 .......... .......... .......... .......... ......--.. ..........

R11preT Q1 .......... .......... .......... .......... ......--.. ..........

R11preT Q1 .......... .......... .......... .......... ......--.. ..........

R11preT Q1 .......... .......... .......... .......... ......--.. ..........

R11preT Q1 .......... .......... .......... .......... ......--.. ..........

R11preT Q1 .......... .......... .......... .......... ......--.. ..........

R11preT Q1 .......... .......... .......... .......... ......--.. ..........

R12preT Q1 .......... .......... .......... .......... ......--.. ..........

R12preT Q2 .......... .......... .......... .......... ......--.. ..........

R12preT Q3 .......... .......... .......... .......... ......--.. ..........

R12preT Q4 .......... .......... .......... .......... ......--.. ..........

R13preT Q1 .......... .......... .......... .......... ......--.. ..........

R13preT Q2 .......... .......... .......... .......... ......--.. ..........

R14preT Q1 .......... .......... .......... .......... ......--.. ..........

R14preT Q2 .......... .......... .......... .......... A.....--.. ..........

R15preT Q1 .......... .......... .......... .......... ......--.. ..........

....|....| ....|....| ....|....| ....|....| ....|....| ....|....|

125 135 145 155 165 175

Consensus CTTTCTTGGA TTAACCCGCT CAATGCCCGG AAATTTGGGC GTGCCCCCGC GAGACTGCTA

R1preT Q1 .......... .......... .......... .......... .......... ..........

R1preT Q2 .......... .......... .......... .......... .......... ..........

R1preT Q3 .......... .......... .......... .......... .......... ..........

R1preT Q4 .......... .......... .......... .......... .......... ..........

R1preT Q5 .......... .......... .......... .......... .......... ..........

R1preT Q6 .......... .......... .......... .......... .......... ..........

R1preT Q7 .......... .......... .......... .......... .......... ..........

R1preT Q8 .......... .......... .......... .......... .......... ..........

R1preT Q9 .......... .......... .......... .......... .......... ..........

R1preT Q10 .......... .......... .......... .......... .......... ..........

R1preT Q11 .......... .......... .......... .......... .......... ..........

R1preT Q12 .......... .......... .......... .......... .......... ..........

R1preT Q13 .......... .......... .......... .......... .......... ..........

R1preT Q14 .......... .......... .......... .......... .......... ..........

R1preT Q15 .......... .......... .......... .......... .......... ..........

R1preT Q16 .......... .......... .......... .......... .......... ..........

R1preT Q17 .......... .......... .......... .......... .......... ..........

R1preT Q18 .......... .......... .......... .......... .......... ..........

R1preT Q19 .......... .......... .......... .......... .......... ..........

R1preT Q20 .......... .......... .......... .......... .......... ..........

R1preT Q21 .......... .......... .......... .......... .......... ..........

R1preT Q22 .......... .......... .......... .......... .......... ..........

R1preT Q23 .......... .......... .......... .......... .......... ..........

R1preT Q24 .......... .......... .......... .......... .......... ..........

R1preT Q25 .......... .......... .......... .......... .......... ..........

R1preT Q26 .......... .......... .......... .......... .......... ..........

R1preT Q27 .......... .......... .......... .......... .......... ..........

R1preT Q28 .......... .......... .......... .......... .......... ..........

R1preT Q29 .......... .......... .......... .......... .......... ..........

R1preT Q30 .......... .......... .......... .......... .......... ..........

R1preT Q31 .......... .......... .......... .......... .......... ..........

R1preT Q32 .......... .......... .......... .......... .......... ..........

R3preT Q1 .......... .C........ .......... .......... .......... ..........

R3preT Q2 .......... .C........ .......... .......... .......... ..........

R4preT Q1 .......... .C........ .......... .......... .......... ..........

R4preT Q2 .......... .C........ .......... .......... .......... ..........

R5preT Q1 .......... .......... .......... .......... .......... A.........

R6preT Q1 .......... .......... .......... .......... .......... A.........

R6preT Q2 .......... .......... .......... .......... .......... A.........

R8preT Q1 .......... .......... .......... .......... .......... ..........

R8preT Q2 .......... .......... .......... .......... .......... ..........

R9preT Q1 .......... .C........ .......... .......... .......... A.........

R9preT Q2 .......... .A........ .......... .......... .......... A.........

R10preT Q1 .......... .......... .......... .......... .......... ..........

R10preT Q2 .......... .......... .......... .......... .......... ..........

R10preT Q3 .......... .......... .......... .......... .......... ..........

R10preT Q4 .......... .......... .......... .......... .......... ..........

R10preT Q5 .......... .......... .......... .......... .......... ..........

R10preT Q6 .......... .......... .......... .......... .......... ..........

R10preT Q7 .......... .......... .......... .......... .......... ..........

R10preT Q8 .......... .......... .......... .......... .......... ..........

R10preT Q9 .......... .......... .......... .......... .......... ..........

R11preT Q1 .......... .......... .......... .......... .......... ..........

R11preT Q2 .......... .......... .......... .......... .......... ..........

R11preT Q3 .......... .......... .......... .......... .......... ..........

R11preT Q4 .......... .......... .......... .......... .......... ..........

R11preT Q5 .......... .......... .......... .......... .......... ..........

R11preT Q6 .......... .......... .......... .......... .......... ..........

R11preT Q7 .......... .......... .......... .......... .......... ..........

R11preT Q8 .......... .......... .......... .......... .......... ..........

R11preT Q9 .......... .......... .......... .......... .......... ..........

R11preT Q1 .......... .......... .......... .......... .......... ..........

R11preT Q1 .......... .......... .......... .......... .......... ..........

R11preT Q1 .......... .......... .......... .......... .......... ..........

R11preT Q1 .......... .......... .......... .......... .......... ..........

R11preT Q1 .......... .......... .......... .......... .......... ..........

R11preT Q1 .......... .......... .......... .......... .......... ..........

R11preT Q1 .......... .......... .......... .......... .......... ..........

R12preT Q1 .......... .......... .......... .......... .......... A.........

R12preT Q2 .......... .......... .......... .......... .......... A.........

R12preT Q3 .......... .......... .......... .......... .......... A.........

R12preT Q4 .......... .......... .......... .......... .......... A.........

R13preT Q1 .......... .......... .......... .......... .......... ..........

R13preT Q2 .......... .......... .......... .......... .......... ..........

R14preT Q1 .......... .......... .......... .......... .......... A.........

R14preT Q2 .......... .......... .......... .......... .......... A.........

R15preT Q1 .......... .......... .......... .......... .......... ..........

....|....| ....|....| ....|....| ....|..

185 195 205 215

Consensus GCCGAGTAGT GTTGGGTCGC GAAAGGCCTT -------

R1preT Q1 .......... .......... .......... -------

R1preT Q2 .......... .......... ..C....... -------

R1preT Q3 ........T. .......... .......... -------

R1preT Q4 ........T. .......... ..C....... -------

R1preT Q5 .......... .......... .......... -------

R1preT Q6 .......... .......... ..C....... -------

R1preT Q7 ........T. .......... .......... -------

R1preT Q8 ........T. .......... ..C....... -------

R1preT Q9 .......... .......... .......... -------

R1preT Q10 ........T. .......... .......... -------

R1preT Q11 .......... .......... ..C....... -------

R1preT Q12 ........T. .......... ..C....... -------

R1preT Q13 .......... .......... .......... -------

R1preT Q14 .......... .......... ..C....... -------

R1preT Q15 ........T. .......... .......... -------

R1preT Q16 ........T. .......... ..C....... -------

R1preT Q17 .......... .......... .......... -------

R1preT Q18 .......... .......... ..C....... -------

R1preT Q19 ........T. .......... .......... -------

R1preT Q20 ........T. .......... ..C....... -------

R1preT Q21 .......... .......... .......... -------

R1preT Q22 .......... .......... ..C....... -------

R1preT Q23 ........T. .......... .......... -------

R1preT Q24 ........T. .......... ..C....... -------

R1preT Q25 .......... .......... .......... -------

R1preT Q26 ........T. .......... .......... -------

R1preT Q27 .......... .......... ..C....... -------

R1preT Q28 ........T. .......... ..C....... -------

R1preT Q29 .......... .......... .......... -------

R1preT Q30 .......... .......... ..C....... -------

R1preT Q31 ........T. .......... .......... -------

R1preT Q32 ........T. .......... ..C....... -------

R3preT Q1 .......... .......... .......... -------

R3preT Q2 .......... .......... .......... -------

R4preT Q1 .......... .......... .......... -------

R4preT Q2 .......... .......... .......... -------

R5preT Q1 .......... .......... .......... -------

R6preT Q1 .......... .......... .......... GTGGTAT

R6preT Q2 .......... .......... .......... GTGGTCT

R8preT Q1 .......... .......... .......... -------

R8preT Q2 .......... .......... .......... -------

R9preT Q1 .......... .......... .......... -------

R9preT Q2 .......... .......... .......... -------

R10preT Q1 .......... .......... .......... -------

R10preT Q2 .......C.. .......... .......... -------

R10preT Q3 .......T.. .......... .......... -------

R10preT Q4 .......... .......... .......... -------

R10preT Q5 .......C.. .......... .......... -------

R10preT Q6 .......T.. .......... .......... -------

R10preT Q7 .......... .......... .......... -------

R10preT Q8 .......C.. .......... .......... -------

R10preT Q9 .......T.. .......... .......... -------

R11preT Q1 .......... .......... .......... -------

R11preT Q2 .......... .......... ..C....... -------

R11preT Q3 .......... .......... .......... -------

R11preT Q4 .......... .......... ..C....... -------

R11preT Q5 .......... .......... .......... -------

R11preT Q6 .......... .......... ..C....... -------

R11preT Q7 .......... .......... .......... -------

R11preT Q8 .......... .......... ..C....... -------

R11preT Q9 .......... .......... .......... -------

R11preT Q1 .......... .......... ..C....... -------

R11preT Q1 .......... .......... .......... -------

R11preT Q1 .......... .......... ..C....... -------

R11preT Q1 .......... .......... .......... -------

R11preT Q1 .......... .......... ..C....... -------

R11preT Q1 .......... .......... .......... -------

R11preT Q1 .......... .......... ..C....... -------

R12preT Q1 .......... .......... ....A.G.C. T------

R12preT Q2 .......... .......... ......G.C. T------

R12preT Q3 .......... .......... ....A.G.C. T------

R12preT Q4 .......... .......... ......G.C. T------

R13preT Q1 .......... .......... .......... -------

R13preT Q2 .......... .......... .......... -------

R14preT Q1 .......... .......... .......... -------

R14preT Q2 .......... .......... .......... -------

R15preT Q1 .......... .......... .......... -------

....|....| ....|....| ....|....| ....|....| ....|....| ....|....|

5 15 25 35 45 55

Consensus GCCATGGCGT TAGTATGAGT GTTGTACAGC CTCCAGGACC CCCCTCCCGG GAGAGCCATA

NR7preT .......... .......... .......... .......... .......... ..........

NR7postT .......... .......... .......... .......... .......... ..........

BT1preT1 .......... .......... .....G.... .......... .......... ..........

BT1preT2 .......... .......... .....G.... .......... .......... ..........

BT1postT1 .......... .......... .....G.... .......... .......... ..........

BT1postT2 .......... .......... .....G.... .......... .......... ..........

BT1postT3 .........A .......... .....G.... .......... .......... ..........

BT1postT4 .........A .......... .....G.... .......... .......... ..........

BT1postT5 .........G .......... .....G.... .......... .......... ..........

BT1postT6 .........G .......... .....G.... .......... .......... ..........

BT2preT1 .........- -...G.AGA. .......... .......... .......... ..........

BT2preT2 .........- -...G.AGA. .......... .......... .......... ..........

BT2postT1 .........- -...G.AGA. .......... .......... .......... ..........

BT2postT2 .........- -...G.AGA. .......... .......... .......... ..........

BT2postT3 .........- -...G.AGA. .......... .......... .......... ..........

BT2postT4 .........- -...G.AGA. .......... .......... .......... ..........

BT3preT1 .......... .G........ .....G.... .......... ...-...... ..........

BT3preT2 .......... .G........ .....G.... .......... ...-...... ..........

BT3postT1 .......... .G........ .....G.... .......... ...-...... ..........

BT3postT2 .......... .G........ .....G.... .......... ...-...... ..........

BT3postT3 .......... .G........ .....G.... .......... ...-...... ..........

BT3postT4 .......... .G........ .....G.... .......... ...-...... ..........

BT3postT5 .......... .G........ .....G.... .......... ...-...... ..........

BT3postT6 .......... .G........ .....G.... .......... ...-...... ..........

BT3postT7 .......... .G........ .....G.... .......... ...-...... ..........

BT3postT8 .......... .G........ .....G.... .......... ...-...... ..........

BT4preT1 .......... .......... .......... .......... ...-...... ..........

BT4postT1 .......... .......... .......... .......... ...-...... ..........

BT4postT2 .......... .......... .......... .......... ...-...... ..........

BT4postT3 .......... .......... .......... .......... ...-...... ..........

BT4postT4 .......... .......... .......... .......... ...-...... ..........

BT5preT1 CT........ .G........ .......... .......... .......... ..........

BT5preT2 CT........ .......... .......... .......... .......... ..........

BT5postT1 CT........ .......... .......... .......... .......... ..........

BT5postT2 CT........ .G........ .......... .......... .......... ..........

BT5postT3 CT........ .......... .......... .......... .......... ..........

BT5postT4 CT........ .......... .......... .......... .......... ..........

BT5postT5 CT........ .G........ .......... .......... .......... ..........

BT5postT6 CT........ .G........ .......... .......... .......... ..........

BT5postT7 CT........ .G........ .......... .......... .......... ..........

BT5postT8 CT........ .......... .......... .......... .......... ..........

******* ** * * ***** **** ********** *** ****** **********

....|....| ....|....| ....|....| ....|....| ....|....| ....|....|

65 75 85 95 105 115

Consensus GTGGTCTGCG GAACCGGTGA GTACACCGGA ATCGCCGGGA TGACCGGGTC CTTTCTTGGA

NR7preT .......... .......... .......... .......... .......... ..........

NR7postT .......... .......... ..T....... .......... C......TC. T..CT.G.A.

BT1preT1 .......... .......... .......... .......... .......... ..........

BT1preT2 .......... .......... .......... .......... .......... ..........

BT1postT1 .......... .......... .......... .......... .......... ..........

BT1postT2 .......... .......... .......... .......... .......... ..........

BT1postT3 .......... .......... .......... .......... .......... ..........

BT1postT4 .......... .......... .......... .......... .......... ..........

BT1postT5 .......... .......... .......... .......... .......... ..........

BT1postT6 .......... .......... .......... .......... .......... ..........

BT2preT1 .......... .......... .......... .......... .......... .GGG......

BT2preT2 .......... .......... .......... .......... .......... .GGG......

BT2postT1 .......... .......... .......... .......... .......... .GGG......

BT2postT2 .......... .......... .......... .......... .......... .GGG......

BT2postT3 .......... .......... .......... .......... .......... .GGG......

BT2postT4 .......... .......... .......... .......... .......... .GGG......

BT3preT1 .......... .......... .......... .......... .......... ..........

BT3preT2 .......... .....T.... .......... .......... .......... ..........

BT3postT1 .......... .......... .......... .......... .......... ..........

BT3postT2 .......... .......... .......... .......... ...T...... ......C...

BT3postT3 .......... .....T.... .......... .......... .......... ..........

BT3postT4 .......... .....T.... .......... .......... ...T...... ......C...

BT3postT5 .......... .......... .......... .......... ...T...... ..........

BT3postT6 .......... .......... .......... .......... .......... ......C...

BT3postT7 .......... .....T.... .......... .......... .......... ......C...

BT3postT8 .......... .....T.... .......... .......... ...T...... ..........

BT4preT1 .......... .......... .......... .......... .......... ..........

BT4postT1 .......... .......... .......... .......... .......... ..........

BT4postT2 .......... .......... .......... .......... .......... ..........

BT4postT3 .....T.... .......... .......... .......... .......... ..........

BT4postT4 .....T.... .......... .......... .......... .......... ..........

BT5preT1 .......... .......... .......... .......... .......... ..........

BT5preT2 .......... .......... .......... .......... .......... ..........

BT5postT1 .......... .......... .......... .......... .......... ..........

BT5postT2 .......... .......... .......... .......... .......... ..........

BT5postT3 .......... .......... .......... .......... .......... ..........

BT5postT4 .......... .......... .......... .......... .......... ..........

BT5postT5 .......... .......... .......... .......... .......... ..........

BT5postT6 .......... .......... .......... .......... .......... ..........

BT5postT7 .......... .......... .......... .......... .......... ..........

BT5postT8 .......... .......... .......... .......... .......... ..........

***** **** ***** **** ** ******* ********** ** *** * * * *

....|....| ....|....| ....|....| ....|....| ....|....| ....|....|

125 135 145 155 165 175

Consensus TTAACCCGCT CAATGCCCGG AAATTTGGGC GTGCCCCCGC GAGACTGCTA GCCGAGTAGT

NR7preT .......... .......... .......... .......... .......... ..........

NR7postT CA........ .......... C......... .......... A......... ..........

BT1preT1 .......... .......... .......... .......... .......... ..........

BT1preT2 .C........ .......... .......... .......... .......... ..........

BT1postT1 .......... .......... .......... .......... .......... ..........

BT1postT2 .C........ .......... .......... .......... .......... ..........

BT1postT3 .C........ .......... .......... .......... .......... ..........

BT1postT4 .......... .......... .......... .......... .......... ..........

BT1postT5 .C........ .......... .......... .......... .......... ..........

BT1postT6 .......... .......... .......... .......... .......... ..........

BT2preT1 .C........ .......... .......... .......... A......... ..........

BT2preT2 .......... .......... .......... .......... A......... ..........

BT2postT1 .C........ .......... .......... .......... A......... ..........

BT2postT2 .......... .......... .......... .......... A......... .......C..

BT2postT3 .C........ .......... .......... .......... A......... .......C..

BT2postT4 .......... .......... .......... .......... A......... ..........

BT3preT1 .......... .......... .......... .......... .......... ..........

BT3preT2 .......... .......... .......... .......... .......... ..........

BT3postT1 .......... .......... .......... .......... .......... ..........

BT3postT2 .......... .......... .......... .......... .......... ..........

BT3postT3 .......... .......... .......... .......... .......... ..........

BT3postT4 .......... .......... .......... .......... .......... ..........

BT3postT5 .......... .......... .......... .......... .......... ..........

BT3postT6 .......... .......... .......... .......... .......... ..........

BT3postT7 .......... .......... .......... .......... .......... ..........

BT3postT8 .......... .......... .......... .......... .......... ..........

BT4preT1 .......... .......... .......... .......... .......... .T........

BT4postT1 .......... .......... .......... .......... .......... .T........

BT4postT2 .......... .......... .......... .......... A......... .T........

BT4postT3 .......... .......... .......... .......... .......... .T........

BT4postT4 .......... .......... .......... .......... A......... .T........

BT5preT1 .......... .......... .......... .......... A......... ..........

BT5preT2 .......... .......... .......... .......... A......... ..........

BT5postT1 .......... .......... .......... .......... A......... ..........

BT5postT2 .......... .......... .......... .......... A......... ..........

BT5postT3 .......... .......... .......... .A........ A......... ..........

BT5postT4 .......... .......... .......... .A........ A......... ..........

BT5postT5 .......... .......... .......... .A........ A......... ..........

BT5postT6 .......... .......... .......... .A........ A......... ..........

BT5postT7 .......... .......... .......... .......... A......... ..........

BT5postT8 .......... .......... .......... .......... A......... ..........

******** ********** ********* * ******** ********* * ***** **

....|....| ....|....| .

185 195

Consensus GTTGGGT-CG CGAAAGGCCT T

NR7preT .......G.. .......... .

NR7postT .......G.. .......... .

BT1preT1 .......-.. .......... .

BT1preT2 .......-.. .......... .

BT1postT1 .......-.. .......... .

BT1postT2 .......-.. .......... .

BT1postT3 .......-.. .......... .

BT1postT4 .......-.. .......... .

BT1postT5 .......-.. .......... .

BT1postT6 .......-.. .......... .

BT2preT1 .......-.. .......... .

BT2preT2 .......-.. .......... .

BT2postT1 .......-.. .......... .

BT2postT2 .......-.. .......... .

BT2postT3 .......-.. .......... .

BT2postT4 .......-.. .......... .

BT3preT1 .......-.. .......... .

BT3preT2 .......-.. .......... .

BT3postT1 .......-.. .......... .

BT3postT2 .......-.. .......... .

BT3postT3 .......-.. .......... .

BT3postT4 .......-.. .......... .

BT3postT5 .......-.. .......... .

BT3postT6 .......-.. .......... .

BT3postT7 .......-.. .......... .

BT3postT8 .......-.. .......... .

BT4preT1 .......-.. .......... .

BT4postT1 .......-.. .......... .

BT4postT2 .......-.. .......... .

BT4postT3 .......-.. .......... .

BT4postT4 .......-.. .......... .

BT5preT1 .....TCG.. .......... .

BT5preT2 .....TCG.. .......... .

BT5postT1 .....TCG.. .......... .

BT5postT2 .....TCG.. .......... .

BT5postT3 .....TCG.. .......... .

BT5postT4 C....TCG.. .......... .

BT5postT5 .....TCG.. .......... .

BT5postT6 C....TCG.. .......... .

BT5postT7 C....TCG.. .......... .

BT5postT8 C....TCG.. .......... .

**** ** ********** *

....|....| ....|....| ....|....| ....|....| ....|....| ....|....|

5 15 25 35 45 55

consensus GCCAT-GGCG TTAGTATGAG TGTTGTACAG CCTCCAGGAC CCCCC-TCCC GGGAGAGCCA

C1 .....-.... .....G.AGA ......G... .......... .....-.... ..........

C2Q1 ....G-.CA. .......... ........GA ....G..... ....---T.. C.........

C2Q2 ....G-.CA. .......... ........GA ....G..... ....---T.. C.........

C3clone 1 .....-.... .......... .......... .......... .....-.... ..........

C3clone 2 .....-.... .......... .......... .......... .....-.... ..........

C4 .....-.... .......... .......... .......... .....-.... ..........

C6Q1 .....-.... .......... ......G... .......... ....--.... ..........

C6Q2 .....-.... .......... ......G... .......... ....--.... ..........

C7Q1 .....T.... .......... .......... .......... .....-.... ..........

C7Q2 .....T.... .......... .......... .......... .....-.... ..........

C7Q3 .....T.... .......... .......... .......... .....-.... ..........

C7Q4 .....T.... .......... .......... .......... .....-.... ..........

C8Q1 .....-.... .......... .......... .......... ....--.... ..........

C8Q2 .....-.... .......... .......... .......... ....--.... ..........

C8Q3 .....-.... .......... .......... .......... ....--.... ..........

C8Q4 .....-.... .......... .......... .......... ....--.... ..........

C9 .....-.... .......... .......... .......... .....C.... ..........

**** * * ***** * ****** * **** ***** **** ** *********

....|....| ....|....| ....|....| ....|....| ....|....| ....|....|

65 75 85 95 105 115

consensus TAGTGGTCTG CGGAACCGGT GAGTACACCG GAATCGCCGG GATGACCGGG TCCTTTCTTG

C1 .......... .......... ....T..... ........A. ..C....... ..........

C2Q1 .......... .......... .......... .......... .......... ..........

C2Q2 .......... .......... .......... .......... .......... ..........

C3clone 1 .......... .......... .......... .......... .......... ..........

C3clone 2 .......... .......... .......... .......... .......... ..........

C4 .......... .......... ....T..... .......... ..C....... ..........

C6Q1 .......... .......... .......... .......... .......... ..........

C6Q2 .......... .......... .......... .......... .......... ..........

C7Q1 .......... .......... .......... .......... .......... ..........

C7Q2 .......... .......... .......... .......... .......... ..........

C7Q3 .......... .......... .......... .......... .......... ..........

C7Q4 .......... .......... .......... .......... .......... ..........

C8Q1 .......... .......... .......... .......... .......... ..........

C8Q2 .......... .......... .......... .......... .......... ..........

C8Q3 .......... .......... .......... .......... .......... ..........

C8Q4 .......... .......... .......... .......... .......... ..........

C9 .......... .......... .......... .......... .......... ..........

********** ********** **** ***** ******** * ** ******* **********

....|....| ....|....| ....|....| ....|....| ....|....| ....|....|

125 135 145 155 165 175

consensus G-ATTAACCC GCTCAATGCC CGGAAATTTG GGCGTGCCCC -CGCGAGACT GCTA-GCCGA

C1 .-........ .G........ T......... .......... G..TAG.C.. ..C.A.....

C2Q1 .-..C..... .......... .......... .......... --........ ..AG-C.A.C

C2Q2 .-..C..... .......... .......... .......... --........ ...G-C.A.C

C3clone 1 .-........ .......... .......... .......... -......... ....-.....

C3clone 2 .-........ .......... .......... .......... -......... ....-.....

C4 .A.CA..... .......... ...C...... .......... -...A..... ....-.....

C6Q1 .-........ .......... .......... .......... -......... ....-.....

C6Q2 .-........ .......... .......... ...A...... -......... ....-.....

C7Q1 .-........ .......... .......... ..AA...... -...A..... ....-.....

C7Q2 .-........ .......... .......... .CAA...... -...A..... ....-.....

C7Q3 --........ .......... .......... ..AAG..... -...A..... ....-.....

C7Q4 .-........ .......... .......... .CAA...... -...A..... ....-.....

C8Q1 .-........ .......... .......... .......... -......... ....-.....

C8Q2 .-..C..... .......... .......... .......... -......... ....-.....

C8Q3 .-..C..... .G........ .......... .......... -......... ....-.....

C8Q4 .-........ .G........ .......... .......... -......... ....-.....

C9 .-........ .......... .......... .C........ -..TAG.C.. ..C.A.....

* * ***** * ******** ** ****** * ***** * * ** ** * *****

....|....| ....|....| ....|...

185 195 205

consensus GTAGT--GTT GGGTGCGCGA AAGGCCTT

C1 AG.ACCC... ...G.TTTCC ........

C2Q1 CG...--... ....T..... ........

C2Q2 CG...--... ....T..... ........

C3clone 1 .....--... ....-..... ........

C3clone 2 .....--... ....-..... ........

C4 .....--... ....-..... ........

C6Q1 .....--... ....-..... ........

C6Q2 .....--... ....-..... ........

C7Q1 .....--... ....-..... ........

C7Q2 .....--... ....-..... ........

C7Q3 .....--... .......... ........

C7Q4 .....--... ....-..... ........

C8Q1 .....--... ....-..... ........

C8Q2 .....--... .......... ........

C8Q3 .....--... .......... ........

C8Q4 .....--... .......... ........

C9 AG.ACCC... ...G.TTTCC ......C.

* *** *** ****** *

....|....| ....|....| ....|....| ....|....| ....|....| ....|....|

5 15 25 35 45 55

Non-respon GCCATGGCGT TAGTATGA-G TGTTGTACAG CCTCCAGGAC CCCCCTCCCG GGAGAGCCAT

Responders .......... ........T. .......... .......... .......... ..........

Controls .......... ........-. .......... .......... .......... ..........

********** ******** * ********** ********** ********** **********

....|....| ....|....| ....|....| ....|....| ....|....| ....|....|

65 75 85 95 105 115

Non-respon AGTGGTCTGC GGAACCGGTG AGTACACCGG AATCGCCGGG ATGACCGGGT CCTTTCTTGG

Responders .......... .......... .......... .......... .......... ..........

Controls .......... .......... .......... .......... .......... ..........

********** ********** ********** ********** ********** **********

....|....| ....|....| ....|....| ....|....| ....|....| ....|....|

125 135 145 155 165 175

Non-respon ATTAACCCGC TCAATGCCCG GAAATTTGGG CGTGCCCCCG CAAGACTGCT AGCCGAGTAG

Responders .......... .......... .......... .......... .G........ ..........

Controls .......... .......... .......... .......... .G........ ..........

********** ********** ********** ********** * ******** **********

....|....| ....|....| ..

185 195

Non-respon TGTTGGGT-C GCGAAAGGCC TT

Responders ........-. .......... ..

Controls ........G. .......... ..

******** * ********** **
